# Analysis of the Structural Characteristics and Psychometric Properties of the Pelvic Floor Bother Questionnaire (PFBQ): A Systematic Review

**DOI:** 10.3390/jcm11237075

**Published:** 2022-11-29

**Authors:** Guadalupe Molina-Torres, Leticia Amiano-López, Marta María Córdoba-Peláez, Alfonso Javier Ibáñez-Vera, Esther Diaz-Mohedo

**Affiliations:** 1 Department of Nursing, Physiotherapy and Medicine, Faculty of Health Sciences, University of Almeria, 04120 Almeria, Spain; 2 Competa Clínic, Calle del Carmen 9, 29754 Competa, Spain; 3 FisioDómina Fisioterapia, Av. Llano de Pablo Picasso 26, 29300 Archidona, Spain; 4 Department of Health Sciences, University of Jaen, Campus las Lagunillas, 23071 Jaen, Spain; 5 Department of Physiotherapy, Faculty of Health Sciences, University of Malaga, Campus Teatinos, 29071 Malaga, Spain

**Keywords:** pelvic floor dysfunctions, questionnaire, validation, psychometric properties, cross-cultural adaptation

## Abstract

Background: The Pelvic Floor Bother Questionnaire is a validated and reliable questionnaire that studies the presence and degree of pelvic floor discomfort, providing a global vision of pelvic floor dysfunction. This questionnaire assesses urinary stress incontinence, urinary urgency, urinary frequency, urge urinary incontinence, pelvic organ prolapses, dysuria, dyspareunia, defecatory dysfunction, fecal incontinence, and the disability it causes to the respondent. Aim: The aim of the present study was to analyze the structural characteristics and psychometric properties of the different versions of the pelvic floor bother questionnaire, as well as the methodological quality, the quality of evidence, and the criteria used for good measurement properties. Methods: A systematic review was carried out in different databases, such as PubMed, SCOPUS, Web of Science, Dialnet, ScienceDirect, and CINAHL, on studies adapting and validating the pelvic floor bother questionnaire in other languages. The data were analyzed taking into account the guidelines of the preferred reporting item statement for systematic reviews and meta-analyses (PRISMA) and following the COSMIN guidelines, considering articles published up to 28 February 2022, and registered in the PROSPERO database. Results: Initially, a total of 443 studies were found, from which a total of four studies were analyzed with regard to structural characteristics and psychometric properties, such as reliability, internal consistency, construct validity, and criterion validity. Conclusions: The different versions of the questionnaires show basic structural characteristics and psychometric properties for the evaluation of patients with pelvic floor dysfunctions. Most of the analyzed versions present criteria for good measurement properties qualified as sufficient, inadequate–adequate methodological quality, and low–moderate quality of evidence.

## 1. Introduction

The pelvic floor (PF) is composed of muscles, ligaments, and fascia that function to support the bladder, reproductive organs, and rectum [1]. This musculature is enclosed within the scaffolding formed by the bones of the pelvis: ilium, ischium, and pubis, which are articulated with the sacrum by two posterior sacroiliac joints and an anterior pubic symphysis joint [2]. The correct function of the muscles and structures that make up the PF is essential, since pelvic floor dysfunction (PFD) can cause symptoms such as: urinary incontinence (UI), whether urgency (UUI), stress (USI) or mixed [3], fecal incontinence (FI), overactive bladder (OB), bladder emptying dysfunction, obstructive defecation syndrome, pelvic organ prolapses (POP) [4] or sexual dysfunctions (dyspareunia, anorgasmia, vaginismus, or vulvodynia), among others [3,5,6]. 

There are risk factors, the best known of which are pregnancy and childbirth, that increase the probability of suffering one of these PFDs [7], although perineal surgeries, obesity, constipation, smoking, lack of knowledge and awareness of the perineal area, and hormonal causes are also behind this symptomatology [8,9]. In reference to prevalence, PFDs are very common, millions of women around the world are affected by this type of problem. Approximately 40% are affected by POP, one in three will experience UI, one in ten will experience UI, and one in ten will experience FI [10] and some may have pain [11], The quality of life of many women is affected to a greater or lesser degree and can affect the social, sexual, and psychological life of women of all ages [12].

In recent years, the use of patient self-reported measures (PROM) [13] has increased considerably both in the field of research and in clinical practice, since they allow the patient to be assessed and the results obtained to be evaluated in a simple way for better planning and monitoring of the patient’s state of health. Thanks to the use of PROMs, we can directly evaluate different subjective aspects of the pathology [14]. Some of these questionnaires include aspects to be assessed as PF symptoms, such as [15,16,17], UI [18,19,20], FI [21], sexual activity [22,23], and quality of life [24].

The Pelvic Floor Bother Questionnaire (PFBQ) is a validated and reliable questionnaire that studies the presence and degree of PF discomfort, providing a global vision of PFD. This questionnaire assesses USI, urinary urgency, urinary frequency, UUI, POP, dysuria, dyspareunia, defecatory dysfunction, FI, and the disability it causes to the respondent. It was developed in 2010 by Peterson et al. in English [25] and subsequently translated and validated in four different languages: Chinese, Turkish, Portuguese-Brazilian, and Arabic [26,27,28,29]. It would be necessary that this pelvic floor assessment tool be adapted and validated in other languages and that the structural characteristics and psychometric properties used in the published versions be taken into account in order to improve future versions. Therefore, the aim of the present study was to analyze the structural characteristics and psychometric properties of the different language versions of the PFBQ, as well as the methodological quality, the quality of evidence, and the criteria used for good measurement properties.

## 2. Materials and Methods

### 2.1. Protocol

A systematic review was carried out considering articles published up to 28 February 2022 and was registered in the PROSPERO database (PROSPERO ID: CRD42022307970) following the recommendations of the PRISMA statement [30] and COSMIN guidelines [31].

### 2.2. Resources and Search

The search was carried out in PubMed, SCOPUS, Web of Science, Dialnet, ScienceDirect, and CINAHL databases. The following MeSH terms were included with the Boolean AND/OR operators: “pelvic floor bother questionnaire” AND “pelvic floor disorders” (“Fecal Incontinence”[Mesh]) OR “Urinary Incontinence”[Mesh]) OR “Pelvic Organ Prolapse”[Mesh]) OR “Pelvic Floor Disorders”[Mesh]) OR “Sexual Dysfunction, Physiological”[Mesh]) OR “Pelvic Girdle Pain”[Mesh]) OR “sexual function” [Title/Abstract]) OR “Prolapse” [Title/Abstract]) AND “Surveys and Questionnaires”[Mesh] AND “Validation”[Title/Abstract] OR “Pelvic Floor Disorders”[Mesh]).

### 2.3. Selection Criteria

The following selection criteria were taken into account in this search: studies that performed a cross-cultural adaptation and validation of the PFBQ in languages other than that of the original publication. The exclusion criteria were: all papers that did not present the results conclusively and that did not include a validation phase.

### 2.4. Selection of Documents

Documents from the different databases were extracted and included in the Rayyan platform [32]. First, the duplicate documents were eliminated, then blinded by two researchers (LAL and MMCP). The documents were selected by title and abstract. In the case of disagreement between the two researchers when selecting the documents, the selection was made by a third researcher (GMT). The documents that were finally selected were obtained in full text to analyze their content and evaluate their inclusion in this review.

### 2.5. Instrument 

The PFBQ is a validated questionnaire that was developed by Cleveland Clinic pelvic floor staff based on clinical interviews and review of commonly used surveys, such as the Urinary Distress Inventory and the Pelvic Floor Distress Inventory (PFDI) and Pelvic Floor Impact Questionnaire (PFIQ) [33,34]. The PFBQ evaluates USI, urinary urgency, urinary frequency, UUI, POP, dysuria, dyspareunia, defecatory dysfunction, fecal incontinence, and the disability generated by the respondent. It consists of 9 items, each scored from 0 to 5, the total score of the questionnaire being between 0 and 45 points, where 0 indicates no discomfort and 45 indicates greater disability. The total score of the questionnaire is multiplied by 20 to obtain a result from 0 to 100 [25].

### 2.6. Synthesis of Results and Data Extraction 

To gather information on the structural characteristics and psychometric properties of each of the versions of the PFBQ, an analysis of the different versions of this questionnaire was carried out.

The methodological quality of each of the versions of a measurement property was analyzed using the risk of bias checklist from the guide of Standards for the selection of health Measurement Instruments (COSMIN) [35,36] whose objective is to facilitate the selection of high-quality PROMs for research and clinical practice [36]. 

The structural characteristics extracted from each version were: title, self-report, year of publication, version, population, sample size, age, sex, characteristics, environment, geographic location, target population, number of subjects - pilot phase, number of subjects per item. On the other hand, the results of the psychometric properties extracted were: test-retest, internal consistency, construct validity, and criterion validity. Subsequently, according to the updated criteria for a good measurement property, the result of each version is evaluated individually for each measurement property and rated as sufficient (+), insufficient (-), or undetermined (?) [36,37]. Finally, the evidence is summarized, and the quality of the evidence is graded according to the approach of Grades of Recommendation, Assessment, Development, and Evaluation (GRADE) [36].

## 3. Results

**Figure 1:**
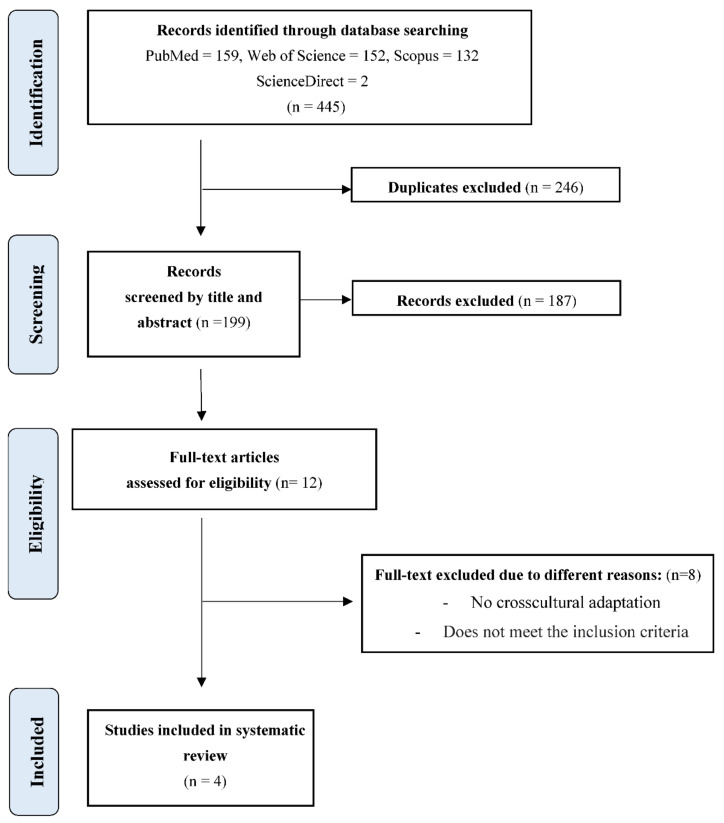
Flowchart for selecting studies based on PRISMA.

After the initial search performed in Pubmed, Scopus, Web of Science, Dialnet, ScienceDirect, and CINAHL databases, as shown in the flow chart of the selected studies (Figure 1), a total of 445 results were found. Excluding duplicates and after selection of papers by title and abstract, 199 were selected, from which 187 were excluded and 12 full-text papers were selected for eligibility, of which eight of them did not meet the inclusion criteria, did not present the results conclusively, did not include a validation phase, or were not a cross-cultural adaptation of PFBQ.

Finally, a total of four versions adapted and validated in languages other than the original were selected: Arabic [28], Chinese [29], Turkish [27], and Portuguese-Brazilian [26]. Then, the structural characteristics of each one of them were analyzed (see Table 1).

In the Arabic version [28] some changes and/or words were introduced to make them culturally acceptable in items 6, 7, and 8. As for item 9, the question “is sexually active” had to be rephrased to “has sexual relations with her husband or male partner”.

Table 2 shows the data corresponding to the psychometric properties of each questionnaire, such as: test-retest, internal consistency, construct validity, and criterion validity.

### 3.1. Structural Validity

None of the versions analyzed assessed the structural validity of the PFBQ except for the Turkish version which included confirmatory factor analysis. The factor analysis tests whether the items of a questionnaire can be classified into different dimensions. When determining the number of dimensions, measurements were taken with a value greater than 1, which subdivided the PFBQ into four dimensions [27].

### 3.2. Internal Consistency

Of the four adaptations of the PFBQ, only Liu et al. [29] and Peterson et al. [26] included this measure using Cronbach’s alpha where values ≥0.70 indicate good internal consistency and values <0.70 are considered low consistency [36]. The Chinese version [29] scored 0.677 and the Portuguese-Brazilian version [26] 0.625, both of which were of low consistency. In addition, only the Turkish version [27] considered the structural validity taken as a requirement in the COSMIN guidelines [31] to determine the level of internal consistency. Therefore, the rest of the versions were rated as “indeterminate”.

On the other hand, neither the Turkish version [27] not the Arabic version [28] evaluated this property because each of the PFBQ questions is focused on a different characteristic, and internal consistency could not be calculated by comparing the scores of individual items with the total score, thus not contributing to the validity of the questionnaire.

### 3.3. Test-Retest Reliability

The four versions calculated test-retest reliability using the intraclass correlation coefficient (ICC) to determine both the total coefficient of the questionnaire and the coefficients by domains. ICC values greater than 0.7 are considered to have acceptable reliability [38]. All the coefficients calculated both globally and by domain exceed 0.7. In the case of the Arabic version, the global score is 0.7 [28], and Doğan et al. [27] obtained the highest score with an ICC = 0.998. The time determined between the first and second test was homogeneous between the Chinese, Turkish, and Portuguese-Brazilian versions, being approximately one week. However, in the Arabic version, a time span of 1–6 weeks was considered. Therefore, according to the criteria for a good measurement of properties, the reliability of all versions was rated as “sufficient”.

### 3.4. Responsiveness

Only the Arabic version [28] and Portuguese-Brazilian version [26] include this measure, the ability of a PROM to detect changes over time in the construct to be measured. Both versions were considered not evaluable because they did not have a hypothesis defined by the review team. Therefore, the methodological quality was inadequate in both versions and the quality of the evidence was low.

### 3.5. Methodological Quality

Methodological quality was assessed according to the COSMIN guidelines criteria [39], which establish, among others, that: “very good” methodological quality requires 7 subjects per item in samples ≥100 people; “adequate” quality requires 5 participants per item in samples ≥100 or 6 subjects per item in samples <100; versions with 5 subjects per item in samples <100 will be rated as “doubtful”; “inadequate” methodological quality is reserved for studies with fewer than 5 subjects per item (see Table 3).

### 3.6. Quality of Evidence

In order to carry out the evaluation of the quality of evidence, data should be classified according to the GRADE approach [40] (high, moderate, low, low, very low evidence), which takes into account the risk of bias, inconsistency, imprecision, and indirectness (see Table 3).

## 4. Discussion

The objective of this review was to analyze the structural characteristics and psychometric properties, as well as to evaluate the methodological quality, quality of evidence, and good measurement properties of the included questionnaires and compare them with the original version [25]. A total of four versions of the PFBQ were included: Arabic [28], Chinese [29], Turkish [27], Portuguese-Brazilian [26].

Regarding the piloting phase, a total of 30 patients were included in the Turkish version [27], 10 patients in Portuguese-Brazilian [26] and Chinese versions [29], and 18 patients in the Arabic version [28], being comparable to this sample in the original version [25], administered to a total of 20 patients. On the other hand, in the validation phase, the version that included more patients was the Portuguese-Brazilian version [26] with a total of 147 patients, and the one with the smallest sample was the Chinese version [29] with 102 patients. The number of patients in the other versions was similar, with 130 in the Turkish version [27] and 130 in the Arabic version [28]. This number of study subjects included in the validation phase is comparable to that included in the original version [25]; 141 patients were included. In order for the questionnaire validation study to be rated as excellent according to the COSMIN guidelines, [31] in this case, the PFBQ consists of nine items, so all the validations of versions would be rated as excellent, since all of them consist of more than 90 sample patients. 

Turkish version only [27] considered structural validity, which included confirmatory factor analysis, and the rest of the versions were rated as indeterminate. Internal consistency was only included in the Chinese [29] and Portuguese-Brazilian versions [26], both of which are of low quality, while the original version [25] obtained a high internal consistency.

Reliability was calculated for all versions [26,27,28,29] by the test-retest. However, the time elapsed between the patients’ responses to the questionnaire rated them as sufficient. In the original version [25], test-retest reliability was very high and the time elapsed between questionnaire administration was one week. 

The psychometric properties measurement error and hypothesis testing were not analyzed in any of the versions of the PFBQ nor in the original version [25]. The internal consistency was only analyzed in the versions of Liu et al. [29] and Peterson et al. [26], resulting in a low internal consistency, contrary to the original version [25] that had good internal consistency. 

### Strengths and Limitations of the Study

This is the first review to analyze the structural characteristics and psychometric properties of this questionnaire in different languages, being a tool used for the assessment of patients with pelvic floor dysfunction. However, the results of this review have limitations that should be taken into account for future versions of the PFBQ as only the Turkish adaptation considered the structural validity (taken as a requirement in the COSMIN guide) to determine the level of internal consistency. Moreover, none of the versions considered the measurement error.

## 5. Conclusions

The PFBQ, focused on the assessment of patients with pelvic floor dysfunction, has been adapted into four languages and each of these versions has criteria for good measurement properties rated as mostly sufficient, inadequate methodological quality, and low-doubtful quality of evidence, taking into consideration the COSMIN guidelines. Different validated instruments, with properties similar to each other and to those of the original questionnaire, are available internationally to health professionals, whether clinicians or researchers. The existence of psychometric properties assures us that the results of research or treatment using any version of the PFBQ are reliable and comparable with each other. We can conclude that the different versions of the PFBQ are valid for use among the Portuguese-Brazilian, Turkish, Arabic, and Chinese-speaking populations and that adaptation of the questionnaire to Spanish and other languages will be necessary for its use in other countries. The psychometric properties and structural characteristics collected in this review should be taken into account to improve future versions.

## Figures and Tables

**Table 1:** Structural characteristics of the questionnaires.

Questionnaire/Author, Year/Version	Population/Sample Size, Age, Group	Affected and Control Group	Setting/Geographical Location	Target Population	Number of Subjects—Phase Pilotage	Number of Subjects per Items
Validation of an Arabic version of the global Pelvic Floor Bother Questionnaire/Bazi et al., 2013 [28]/Arabic version	*n* = 130 patients Age.- AG: 50.14 ± 12.64 CG: 45.54 ± 11.19 Parity.- AF: 2.72 ± 1.96 CG: 1.83 ± 1.78	AG: 65 CG: 65	American University of Beirut medical center (AUBMC), Lebanon	UI or POP	18	14.44
Reliability and validity of a Turkish version of the Global Pelvic Floor Bother Questionnaire/Doğan et al., 2016 [27]/Turkish version	*n* = 131 patients. Age.- 46.83 ± 11.19		Department of Obstetrics and Gynecology at Abant Izzet Baysal University in Bolu, Turkey	SUI, UF, UU, VD, POP, OF, FI, or dyspareunia.	30	14.55
Validation of the Brazilian Portuguese version of the pelvic floor bother questionnaire/Peterson et al., 2019 [26]/Portuguese version	*n* = 147 Age 60.49 ± 12.02 Median parity 2 (0–15)		Urogynecology sector, Gynecology Discipline and the Anorectal Physiology sector, Discipline of Digestive System Surgery and Colorectal Surgery at the Clinics Hospital of University of São Paulo Medical School (HCFMUSP), São Paulo, Brazil	PFD	10	16.33
Validation of a Chinese version for the global Pelvic Floor Bother Questionnaire/Liu et al., 2022 [29]/Chinese version	*n* = 102 patients Age.- CG: 32.84 ± 9.382 AG: 30.92 ± 7.022 Parity.- CG: 1.31 ± 0.735 AG: 1.53 ± 0.731	AG: 51 CG: 51	Department of Rehabilitation Medicine, Third Xiangya Hospital of Central South University, Hunan, China	PFD	10	11.33

AG: Affected Group, CG: Control Group, PFD: Pelvic Floor Dysfunction, UI: urinary incontinence, POP: pelvic organ prolapse, SUI: stress urinary incontinence, UF: urinary frequency, UU: urinary urgence, VD: voiding difficulty, OF: obstructed defecation, FI: fecal incontinence.

**Table 2:** Psychometrics properties of the questionnaires.

Study/Version	Test-Retest Reliability	Internal Consistency	Construct Validity				Criterion Validity		
			**Discriminant Validity** **r *(p*-Value)**						
Bazi et al., 2013 [28]/ Arabic version	ICC = 0.7 Q1: 0.812 (0.623–0.911) Q2: 0.962 (0.917–0.983) Q3: 0.774 (0.558–0.892) Q4: 0.967 (0.927–0.985) Q5. 0.951 (0.894–0.978) Q6. 0.976 (0.948–0.989) Q7. 0.972 (0.938–0.987) Q8: 0.900 (0.791–0.954) Q9: 0.844 (0.683–0.927)	NR	NR				NR		
Doğan et al., 2016 [27]/ Turkish version	ICC = 0.998 Q1 = 0.981 (*p* < 0.0001) Q2 = 0.985 (*p* < 0.0001) Q3 = 0.993 (*p* < 0.0001) Q4 = 0.979 (*p* < 0.0001) Q5 = 0.920 (*p* < 0.0001) Q6 = 0.985 (*p* < 0.0001) Q7 = 0.993 (*p* < 0.0001) Q8 = 0.990 (*p* < 0.0001) Q9 = 0.992 (*p* < 0.0001)	NR	NR					PFDI-20	PFIQ-7
							GPFBQ	r = 0.860 *p* = 0.000 *	r = 0.802 *p* = 0.000 *
							PFDI-20		r = 0.814 *p* = 0.000 *
							* *p* < 0.0001		
Peterson, T. et al., 2019 [26]/ Brazilian version	ICC = 0.981 Q1: 0.968 (0.923–1.00) Q2: 0.920 (0.851–0.989) Q3: 0.951 (0.896–1.00) Q4: 0.970 (0.929–1.00) Q5: 0.895 (0.795–0.995) Q6: 0.984 (0.953–1.00) Q7: 0.970 (0.929–1.00) Q8: 0.971 (0.930–1.00) Q9: 1.00 (1.00–1.00)	Cronbach’s α = 0.625	NR				NR		
Liu, Z. et al., 2022 [29]/ Chinese version	ICC = 0.938 Q1: 0.981 (0.960–0.991) Q2: 0.717 (0.475–0.858) Q3: 0.849 (0.700–0.927) Q4: 0.847 (0.696–0.926) Q5: 0.865 (0.729–0.935) Q6: 0.935 (0.865–0.969) Q7: 0.774 (0.568–0.889) Q8: 0.950 (0.896–0.977) Q9: 0.792 (0.599–0.898) Total Score: 0.938 (0.870–0.971)	Cronbach’s α = 0.677 (whole questionnaire) Cronbach’s α = 0.649 (urinary symptoms)	PFBQ-related item	Control women (*n* = 51)	PFD patients (*n* = 51)	*p* value	NR		
			SUI	0 (0–3)	2 (0–5)	<0.001 ^a^			
			UF	0 (0–3)	0 (0–5)	<0.001 ^a^			
			UU	0 (0–3)	1 (0–5)	<0.001 ^a^			
			UUI	0 (0–3)	0 (0–5)	0.004 ^a^			
			VD	0 (0–3)	0 (0–5)	0.269 ^a^			
			POP	0 (0–3)	1 (0–5)	<0.001 ^a^			
			OD	0 (0–3)	1 (0–5)	<0.001 ^a^			
			FI	0 (0–3)	0 (0–5)	0.006 ^a^			
			Dyspareunia	1 (0–3)	2 (0–4)	0.010 ^a^			

NR: not reported, SUI: stress urinary incontinence, UF: urinary frequency, UU: urinary urgence, UUI: Urgency Urinary incontinence, VD: voiding difficulty, POP: pelvic organ prolapse, OD: obstructed defecation, FI: fecal incontinence. * *p* < 0.0001, ^a^ Indicates *p* values obtained with Mann–Whitney U tests.

**Table 3:** Analysis of the rating of the psychometric properties, methodological quality and quality of evidence.

PROM	Version	Structural Validity (Rating)	Internal Consistency (Rating)	Reliability (Rating)	Measurement Error (Rating)	Hypotheses Testing (Rating)	Responsiveness (Rating)
Bazi et al, 2013 [28]	Arabic	NR	NE	Sufficient	NR	NR	NE
Methodological quality Risk of bias		NR	Inadequate	Doubtful	NR	NR	Inadequate
Quality of evidence		NR	Low	Moderate	NR	NR	Low
Doğan et al, 2016 [27]	Turkish	Indeterminate	NE	Sufficient	NR	NR	NR
Methodological quality Risk of bias		Doubtful	Inadequate	Doubtful	NR	NR	NR
Quality of evidence		NE	Low	Moderate	NR	NR	NR
Peterson et al, 2019 [26]	Brazilian Portuguese	NR	NE	Sufficient	NR	NR	NE
Methodological quality Risk of bias		NR	NE	Adequate	NR	NR	Inadequate
Quality of evidence		NR	NR	Moderate	NR	NR	Low
Liu et al, 2022 [29]	Chinese	NR	NE	Sufficient	NR	NR	NR
Methodological quality Risk of bias		NR	NE	Adequate	NR	NR	NR
Quality of evidence		NR	NR	Moderate	NR	NR	NR

NR: not reported; NE: not evaluable.

## Data Availability

Not applicable.
